# Vacuum Landscaping: Cause of Nonlocal Influences without Signaling

**DOI:** 10.3390/e20060458

**Published:** 2018-06-13

**Authors:** Gerhard Grössing, Siegfried Fussy, Johannes Mesa Pascasio, Herbert Schwabl

**Affiliations:** Austrian Institute for Nonlinear Studies, Akademiehof, Friedrichstrasse 10, 1010 Vienna, Austria

**Keywords:** Schrödinger equation, de Broglie–Bohm theory, nonequilibrium thermodynamics, zero-point field, 03.65.-w, 03.65.Ta, 05.40.-a, 05.70.Ln

## Abstract

In the quest for an understanding of nonlocality with respect to an appropriate ontology, we propose a “cosmological solution”. We assume that from the beginning of the universe each point in space has been the location of a scalar field representing a zero-point vacuum energy that nonlocally vibrates at a vast range of different frequencies across the whole universe. A quantum, then, is a nonequilibrium steady state in the form of a “bouncer” coupled resonantly to one of those (particle type dependent) frequencies, in remote analogy to the bouncing oil drops on an oscillating oil bath as in Couder’s experiments. A major difference to the latter analogy is given by the nonlocal nature of the vacuum oscillations. We show with the examples of double- and *n*-slit interference that the assumed nonlocality of the distribution functions alone suffices to derive the de Broglie–Bohm guiding equation for *N* particles with otherwise purely classical means. In our model, no influences from configuration space are required, as everything can be described in 3-space. Importantly, the setting up of an experimental arrangement limits and shapes the forward and osmotic contributions and is described as vacuum landscaping.

## 1. Introduction: Quantum Mechanics without Wavefunctions

“Emergent Quantum Mechanics” stands for the idea that quantum mechanics is based on a more encompassing deeper level theory. This counters the traditional belief, usually expressed in the context of orthodox Copenhagen-type quantum mechanics, that quantum theory is an “ultimate” theory whose main features will prevail for all time and will be applicable to all questions of physics. Note, for example, that, even in more recent approaches to spacetime, the concept of an “emergent spacetime” is introduced as a description even of space and time emerging from basic quantum mechanical entities. This, of course, need not be so, considering the fact that there is “plenty of room at the bottom,” i.e., as Feynman implied, between present-day resolutions and minimally possible times and distances, which could in principle be way below resolutions reasonably argued about in present times (i.e., on Planck scales).

One of the main attractive features of the de Broglie–Bohm interpretation of the quantum mechanical formalism, and of Bohmian mechanics as well, lies in the possibility to extend its domain into space and/or time resolutions where modified behaviors different from quantum mechanical ones may be expected. In other words, there may be new physics involved that would require an explicitly more encompassing theory than quantum mechanics, i.e., a deeper level theory. Our group’s approach, which we pursued throughout the last 10 years, is characterized by the search for such a theory under the premise that even for nonrelativistic quantum mechanics, the Schrödinger equation cannot be an appropriate starting point, since the wavefunction is still lacking a firm theoretical basis and its meaning is generally not agreed upon.

For a similar reason, also the de Broglie–Bohm theory cannot be our starting point, as it is based on the Schrödinger equation and the use of the wavefunction to begin with. Rather, we aim at an explicit ansatz for a deeper level theory without wavefunctions, from which the Schrödinger equation, or the de Broglie–Bohm guiding equation, can be derived. We firmly believe that we have accomplished this and we can now proceed to study consequences of the approach beyond orthodox expectations.

Throughout recent years, apart from our own model, several approaches to a quantum mechanics without wavefunctions have been proposed [1,2,3,4,5]. These refer to “many classical worlds” that provide Bohm-type trajectories with certain repulsion effects. From our realistic point of view, the true ontologies of these models, however, do not become apparent. So let us turn to our model. As every physical theory is based on metaphysical assumptions, we must make clear what our assumptions are. They are as follows.

We propose a “cosmological solution” in that the Big Bang, or any other model explaining the apparent expansion of the universe, is essentially related to the vacuum energy (The latter may constitute what is called the dark energy, but we do not need to specify this here). We assume that from the beginning of the universe each point in space has been the location of a scalar field representing a zero-point vacuum energy that vibrates at a vast range of different frequencies across the whole universe. More specifically, we consider the universe as an energetically open system where the vacuum energy not only drives expansion, but also each individual “particle” oscillation ω=E/ℏ in the universe. In order to maintain a particular frequency, any such oscillator must be characterized by a throughput of energy external to it. In this regard, we have time and again employed the analogy of Couder’s experiments with bouncing oil drops on a vibrating bath [6,7,8,9,10,11]: The bouncer/particle is always in resonant interaction with a relevant environment.

Our model, though also largely classical, has a very different ontology from the “many classical worlds” one. We consider *one* “superclassical” world instead: a purely classical world plus “cosmological nonlocality,” i.e., a nonlocal bath for every oscillator/particle due to the all-pervading vacuum energy, which—mostly in the context of quantum mechanics—is called the zero-point energy. Thus, it is the one classical world together with the fluctuating environment related to the vacuum energy that enters our definition of a quantum as an emergent system. The latter consists of a bouncer and an undulatory/wave-like nonlocal environment defined by proper boundary conditions (As an aside we note that this is not related to de Broglie’s “nonlinear wave mechanics” [12], as there the nonlinear wave, with the particle as soliton-like singularity, is considered as one ontic entity. In our case, however, we speak of two separate, though synchronous elements: local oscillators and generally nonlocal oscillating fields).

In previous work, we have shown how the Schrödinger equation can be derived from a nonequilibrium sub-quantum dynamics [13,14,15,16], where in accordance with the model sketched above the particle is considered as a steady state with a constant throughput of energy. This, then, leads to the two-momentum approach to emergent quantum mechanics which shall be outlined in the next section.

## 2. The Two-Momenta Approach to Emergent Quantum Mechanics

We consider the empirical fact that each particle of nature is attributed an energy E=ℏω as one of the essential features of quantum systems (We have also presented a classical explanation for this relation from our sub-quantum model [17], but do not need to use the details for our present purposes). Oscillations, characterized by some typical angular frequency ω, are described as properties of off-equilibrium steady-state systems. ”Particles” can then be assumed to be dissipative systems maintained in a nonequilibrium steady-state by a permanent throughput of energy, or heat flow, respectively. The heat flow must be described by an external kinetic energy term. Then the energy of the total system, i.e., of the particle and it’s thermal context, becomes

(1) $$E_{\mathrm{tot}}=\hbar \omega +\frac{{\left(\delta p\right)}^{2}}{2m}\;,$$

where δp is an additional, fluctuating momentum component of the particle of mass *m*.

We assume that an effect of said thermal context is given by detection probability distributions, which are wave-like in the particle’s surroundings. Thus, the detection probability density P(x,t) is considered to coincide with a classical wave’s intensity $I\left(x,t\right)=R^{2}\left(x,t\right),$ with R(x,t) being the wave’s real-valued amplitude

(2) $$P\left(x,t\right)=R^{2}\left(x,t\right)\;,\;\mathrm{with}\;\mathrm{normalization}\;\int P\;d^{n}x=1\;.$$

In [13], we combine some results of nonequilibrium thermodynamics with classical wave mechanics. We propose that the many microscopic degrees of freedom associated with the hypothesized sub-quantum medium can be recast into the emergent macroscopic properties of the wave-like behavior on the quantum level. Thus, for the relevant description of the total system, one no longer needs the full phase space information of all microscopic entities, but only the emergent particle coordinates.

For implementation, we model a particle as being surrounded by a heat bath, i.e., a reservoir that is very large compared to the small dissipative system, such that that the momentum distribution in this region is given by the usual Maxwell–Boltzmann distribution. This corresponds to a “thermostatic” regulation of the reservoir’s temperature, which is equivalent to the statement that the energy lost to the thermostat can be regarded as heat. Thus, one can formulate a *proposition of emergence* [13] providing the equilibrium-type probability (density) ratio

(3) $$\frac{P(x,t)}{P(x,0)}=e^{-\frac{\Delta Q(t)}{kT}}\;,$$

with *k* being Boltzmann’s constant, *T* the reservoir temperature, and ΔQ(t) the heat that is exchanged between the particle and its environment.

Equations (1)–(3) are the only assumptions necessary to derive the Schrödinger equation from (modern) classical mechanics. We need to employ only two additional well-known results. The first is given by Boltzmann’s formula for the slow transformation of a periodic motion (with period τ=2π/ω) upon application of a heat transfer ΔQ. This is needed as we deal with an oscillator of angular frequency ω in a heat bath *Q*, and a change in the vacuum surroundings of the oscillator will come as a heat transfer ΔQ. The latter is responsible for a change δS of the action function *S* representing the effect of the vacuum’s “zero-point” fluctuations. With the action function $S=\int \left(E_{\mathrm{kin}}-V\right)\;dt$, the relation between heat and action was first given by Boltzmann [18],

(4) ΔQ(t)=2ω[δS(t)−δS(0)].

Finally, the requirement that the average kinetic energy of the thermostat equals the average kinetic energy of the oscillator is given, for each degree of freedom, by

(5) $$\frac{kT}{2}=\frac{\hbar \omega }{2}\;.$$

Combining these two results, Equations (4) and (5), with Equation (3), one obtains

(6) $$P\left(x,t\right)=P\left(x,0\right)e^{-\frac{2}{\hbar }\left[\delta S\left(x,t\right)-\delta S\left(x,0\right)\right]}\;,$$

from which follows the expression for the momentum fluctuation δp of Equation (1) as

(7) $$\delta p\left(x,t\right)=\nabla \left(\delta S\left(x,t\right)\right)=-\frac{\hbar }{2}\frac{\nabla P(x,t)}{P(x,t)}\;.$$

This, then, provides the additional kinetic energy term for one particle as

(8) $$\delta E_{\mathrm{kin}}=\frac{1}{2m}\nabla \left(\delta S\right)\cdot \nabla \left(\delta S\right)=\frac{1}{2m}{\left(\frac{\hbar }{2}\frac{\nabla P}{P}\right)}^{2}\;.$$

Thus, writing down a classical action integral for j=N particles in *m*-dimensional space, including this new term for each of them, yields (with external potential *V*)

(9) $$A=\int L\;d^{m}x\;dt=\int P\left[\frac{\partial S}{\partial t}+\sum_{j=1}^{N}\frac{1}{2m_{j}}\nabla_{j}S\cdot \nabla_{j}S+\sum_{j=1}^{N}\frac{1}{2m_{j}}{\left(\frac{\hbar }{2}\frac{\nabla_{j}P}{P}\right)}^{2}+V\right]\;d^{m}x\;dt\;$$

where the probability density $P=P(x_{1},x_{2},\ldots ,x_{N},t)$.

With the definition of forward and osmotic velocities, respectively,

(10) $$v_{j}:=\frac{p_{j}}{m_{j}}=\frac{\nabla_{j}S}{m_{j}}\;\mathrm{and}\;u_{j}:=\frac{\delta p_{j}}{m_{j}}=-\frac{\hbar }{2m_{j}}\frac{\nabla_{j}P}{P},$$

one can rewrite Equation (9) as

(11) $$A=\int L\;d^{m}x\;dt=\int P\left[\frac{\partial S}{\partial t}+V+\sum_{j=1}^{N}\frac{m_{j}}{2}v_{j}^{2}+\sum_{j=1}^{N}\frac{m_{j}}{2}u_{j}^{2}\right]\;d^{m}x\;dt\;.$$

This can be considered as the basis for our approach with two momenta, i.e., the forward momentum mv and the osmotic momentum mu, respectively. At first glance, the Lagrangian in Equation (11) looks completely classical, with two kinetic energy terms per particle instead of one. However, due to the particular nature of the osmotic momentum as given in Equation (10), nonlocal influences are introduced: even at long distances away from the particle location, where the particle’s contribution to *P* is practically negligibly small, the expression of the form $\frac{\nabla_{j}P}{P}$ may be large and affects immediately the whole fluctuating environment. This is why the osmotic variant of the kinetic energy makes all the difference to the usual classical mechanics, or, in other words, is the basis for quantum mechanics.

Introducing now the Madelung transformation

(12) $$\psi =R\;e^{\frac{i}{\hbar }S}\;$$

where $R=\sqrt{P}$ as in Equation (2), one has, with bars denoting averages,

(13) $$\bar{{\left|\frac{\nabla_{j}\psi }{\psi }\right|}^{2}}:=\int \;d^{m}x\;dt{\left|\frac{\nabla_{j}\psi }{\psi }\right|}^{2}=\bar{{\left(\frac{1}{2}\frac{\nabla_{j}P}{P}\right)}^{2}}+\bar{{\left(\frac{\nabla_{j}S}{\hbar }\right)}^{2}}\;,$$

and one can rewrite Equation (9) as

(14) $$A=\int L\;d^{m}x\;dt=\int \;d^{m}x\;dt\left[{\left|\psi \right|}^{2}\left(\frac{\partial S}{\partial t}+V\right)+\sum_{j=1}^{N}\frac{\hbar^{2}}{2m_{j}}{\left|\nabla_{j}\psi \right|}^{2}\right]\;.$$

Thus, with the identity ${\left|\psi \right|}^{2}\frac{\partial S}{\partial t}=-\frac{i\hbar }{2}\left(\psi^{*}\dot{\psi }-{\dot{\psi }}^{*}\psi \right)$, one obtains the familiar Lagrange density

(15) $$L=-\frac{i\hbar }{2}\left(\psi^{*}\dot{\psi }-{\dot{\psi }}^{*}\psi \right)+\sum_{j=1}^{N}\frac{\hbar^{2}}{2m_{j}}\nabla_{j}\psi \cdot \nabla_{j}\psi^{*}+V\psi^{*}\psi \;,$$

from which by the usual procedures one arrives at the *N*-particle Schrödinger equation

(16) $$i\hbar \frac{\partial \psi }{\partial t}=\left(-\sum_{j=1}^{N}\frac{\hbar^{2}}{2m_{j}}\nabla_{j}^{2}+V\right)\psi \;.$$

Note also that from Equation (9) one obtains upon variation in *P* the modified Hamilton–Jacobi equation familiar from the de Broglie–Bohm interpretation, i.e.,

(17) $$\frac{\partial S}{\partial t}+\sum_{j=1}^{N}\frac{{\left(\nabla_{j}S\right)}^{2}}{2m_{j}}+V\left(x_{1},x_{2},\cdots ,x_{N},t\right)+U\left(x_{1},x_{2},\ldots ,x_{N},t\right)=0\;$$

where *U* is known as the “quantum potential”

(18) $$U\left(x_{1},x_{2},\ldots ,x_{N},t\right)=\sum_{j=1}^{N}\frac{\hbar^{2}}{4m_{j}}\left[\frac{1}{2}{\left(\frac{\nabla_{j}P}{P}\right)}^{2}-\frac{\nabla_{j}^{2}P}{P}\right]=-\sum_{j=1}^{N}\frac{\hbar^{2}}{2m_{j}}\frac{\nabla_{j}^{2}R}{R}\;.$$

Moreover, with the definitions of $u_{j}$ in Equation (10) one can rewrite *U* as

(19) $$U=\sum_{j=1}^{N}\left[\frac{m_{j}u_{j}^{2}}{2}-\frac{\hbar }{2}\left(\nabla_{j}\cdot u_{j}\right)\right]\;.$$

However, as was already pointed out in [13], with the aid of Equations (4) and (6), $u_{j}$ can also be written as

(20) $$u_{j}=\frac{1}{2\omega_{j}m_{j}}\nabla_{j}Q\;,$$

which thus explicitly shows its dependence on the spatial behavior of the heat flow δQ. Insertion of Equation (20) into Equation (19) then provides the thermodynamic formulation of the quantum potential as

(21) $$U=\sum_{j=1}^{N}\frac{\hbar^{2}}{4m_{j}}\left[\frac{1}{2}{\left(\frac{\nabla_{j}Q}{\hbar \omega_{j}}\right)}^{2}-\frac{\nabla_{j}^{2}Q}{\hbar \omega_{j}}\right]\;.$$

**Figure 1:**
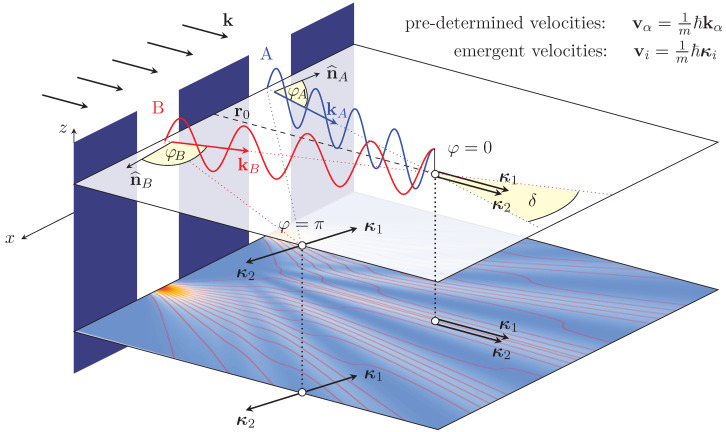
Scheme of interference at a double-slit. Considering an incoming beam of electrons with wave number k impinging on a wall with two slits, two beams with wave numbers $k_{A}$ and $k_{B}$, respectively, are created, which one may denote as “pre-determined” velocities $v_{\alpha }=\frac{1}{m}\hbar k_{\alpha },\;\alpha =A\;\mathrm{or}\;B.$ Taking into account the influences of the osmotic momentum field mu, one has to combine all the velocities/momenta at a given point in space and time in order to compute the resulting, or emergent, velocity/momentum field $v_{i}=\frac{1}{m}\hbar \kappa_{i},\;i=1\;\mathrm{or}\;2$. This, then, provides the correct intensity distributions and average trajectories (lower plane).

As in our model particles and fields are dynamically interlocked, it would be highly misleading to picture the quantum potential in a manner similar to the classical scenario of particle plus field, where the latter can be switched on and off like an ordinary potential. Contrariwise, in our case the particle velocities/momenta must be considered as *emergent*. One can illustrate this with the situation in double-slit interference (Figure 1). Considering an incoming beam of, say, electrons with wave number k impinging on a wall with two slits, two beams with wave numbers $k_{A}$ and $k_{B}$, respectively, are created, which one may denote as “pre-determined” quantities, resulting also in pre-determined velocities $v_{\alpha }=\frac{1}{m}\hbar k_{\alpha }$, α=AorB.

However, if one considers that the electrons are not moving in empty space, but in an undulatory environment created by the ubiquitous zero-point field “filling” the whole experimental setup. One has to combine all the velocities/momenta at a given point in space and time in order to compute the resulting, or emergent, velocity/momentum field $v_{i}=\frac{1}{m}\hbar \kappa_{i}$, i=1or2 (Figure 1), where *i* is a bookkeeping index not necessarily related to the particle coming from a particular slit [19]. The relevant contributions other than the particle’s forward momentum mv originate from the osmotic momentum mu. The latter is well known from Nelson’s stochastic theory [20], but its identical form has been derived by one of us from an assumed sub-quantum nonequilibrium thermodynamics [13,21] as it was described above. As shall be shown in the next section, our model also provides an understanding and deeper-level explanation of the microphysical, causal processes involved, i.e., of the guiding law [22] of the de Broglie–Bohm theory.

## 3. Derivation of the De Broglie–Bohm Guiding Equation for *N* Particles

Consider at first one particle in an *n*-slit system. In quantum mechanics, as well as in our emergent quantum mechanics approach, one can write down a formula for the total intensity distribution *P* which is very similar to the classical formula. For the general case of *n* slits, it holds with phase differences $\varphi_{ii^{\prime }}=\varphi_{i}-\varphi_{i^{\prime }}$ between the slits *i*, $i^{\prime }$ that

(22) $$P=\sum_{i=1}^{n}\left(P_{i}+\sum_{i^{\prime }=i+1}^{n}2R_{i}R_{i^{\prime }}\cos\varphi_{ii^{\prime }}\right)$$

where the phase differences are defined over the whole domain of the experimental setup. As in our model, the “particle” is actually a bouncer in a fluctuating wave-like environment, i.e., analogously to the bouncers of the Couder experiments, one does have some (e.g., Gaussian) distribution, with its center following the Ehrenfest trajectory in the free case, but one also has a diffusion to the right and to the left of the mean path, which is only due to that stochastic bouncing. Thus, the total velocity field of our bouncer in its fluctuating environment is given by the sum of the forward velocity v and the respective osmotic velocities $u_{L}$ and $u_{R}$ to the left and the right. As for any direction α the osmotic velocity $u_{\alpha }=\frac{\hbar }{2m}\frac{\nabla P}{P}$ does not necessarily fall off with the distance, one has long effective tails of the distributions which contribute to the nonlocal nature of the interference phenomena [23]. In sum, one has three distinct velocity (or current) channels per slit in an *n*-slit system.

We have previously shown [19,24] how one can derive the Bohmian guidance formula from our two-momentum approach. Introducing classical wave amplitudes $R(w_{i})$ and generalized velocity field vectors $w_{i}$, which represent either a forward velocity v or an osmotic velocity u in the direction transversal to v, we calculate the phase-dependent amplitude contributions of the total system’s wave field projected on one channel’s amplitude $R(w_{i})$ at the point (x,t) in the following way. We define a *relational intensity* $P(w_{i})$ as the local wave intensity $P(w_{i})$ in each channel (i.e., $w_{i}$), recalling that there are 3 velocity channels per slit: $u_{L}$, $u_{R}$, and v. The sum of all relational intensities, then, is the total intensity, i.e., the total probability density. In an *n*-slit system, we thus obtain for the relational intensities and the corresponding currents, respectively, i.e., for each channel component i,

(23) $$\begin{array}{cc} P(w_{i}) & =R\left(w_{i}\right){\hat{w}}_{i}\cdot \sum_{i^{\prime }=1}^{3n}{\hat{w}}_{i^{\prime }}R\left(w_{i^{\prime }}\right) \end{array}$$

(24) $$\begin{array}{cc} J(w_{i}) & =w_{i}P\left(w_{i}\right),\;i=1,\ldots ,3n \end{array}$$

with unit vectors ${\hat{w}}_{i}$ and

(25) $$\cos\varphi_{ii^{\prime }}:={\hat{w}}_{i}\cdot {\hat{w}}_{i^{\prime }}\;.$$

Consequently, the total intensity and current of our field read as

(26) $$\begin{array}{cc} P_{\mathrm{tot}}= & \sum_{i=1}^{3n}P\left(w_{i}\right)={\left(\sum_{i=1}^{3n}{\hat{w}}_{i}R\left(w_{i}\right)\right)}^{2} \end{array}$$

(27) $$\begin{array}{cc} J_{\mathrm{tot}}= & \sum_{i=1}^{3n}J\left(w_{i}\right)=\sum_{i=1}^{3n}w_{i}P\left(w_{i}\right), \end{array}$$

leading to the *emergent total velocity*

(28) $$v_{\mathrm{tot}}=\frac{J_{\mathrm{tot}}}{P_{\mathrm{tot}}}=\frac{\sum_{i=1}^{3n}w_{i}P\left(w_{i}\right)}{\sum_{i=1}^{3n}P\left(w_{i}\right)}\;,$$

which represents the *probability flux lines.*

In [16,19], we have shown with the example of n=2, i.e., a double-slit system, that Equation (28) can equivalently be written in the form

(29) $$v_{\mathrm{tot}}=\frac{R_{1}^{2}v_{1}+R_{2}^{2}v_{2}+R_{1}R_{2}\left(v_{1}+v_{2}\right)\cos\varphi +R_{1}R_{2}\left(u_{1}-u_{2}\right)\sin\varphi }{R_{1}^{2}+R_{2}^{2}+2R_{1}R_{2}\cos\varphi }\;.$$

The trajectories or streamlines, respectively, are obtained according to $\dot{x}=v_{\mathrm{tot}}$ in the usual way by integration. As we have first shown in [16], by re-inserting the expressions for forward and osmotic velocities, respectively, i.e.,

(30) $$v_{i}=\frac{\nabla S_{i}}{m}\;,\;u_{i}=-\frac{\hbar }{m}\frac{\nabla R_{i}}{R_{i}}\;,$$

one immediately identifies Equation (29) with the Bohmian guidance formula. Naturally, employing the Madelung transformation for each slit α (α=1 or 2),

(31) $$\psi_{\alpha }=R_{\alpha }e^{iS_{\alpha }/\hbar },$$

so $P_{\alpha }=R_{\alpha }^{2}={\left|\psi_{\alpha }\right|}^{2}=\psi_{\alpha }^{*}\psi_{\alpha }$, with $\varphi =(S_{1}-S_{2})/\hbar$. Recalling the usual trigonometric identities such as $\cos\varphi =\frac{1}{2}\left(e^{i\varphi }+e^{-i\varphi }\right)$, one can rewrite the total average current immediately in the usual quantum mechanical form as

(32) Jtot=Ptotvtot=(ψ1+ψ2)∗(ψ1+ψ2)121m−iℏ∇(ψ1+ψ2)(ψ1+ψ2)+1miℏ∇(ψ1+ψ2)∗(ψ1+ψ2)∗=−iℏ2mΨ∗∇Ψ−Ψ∇Ψ∗=1mReΨ∗(−iℏ∇)Ψ

where $P_{\mathrm{tot}}=|\psi_{1}+\psi_{2}{|}^{2}=:{\left|\Psi \right|}^{2}$.

Equation (28) has been derived for one particle in an *n*-slit system. However, for the spinless particles obeying the Schrödinger equation, it is easy to extend this derivation to the many-particle case (As we do not yet have a relativistic model involving spin, our results for the many-particle case cannot account for the difference in particle statistics, i.e., for fermions or bosons. This will be a task for future work). Due to the purely additive terms in the expressions for the total current and total probability density, respectively, also for *N* particles, Equations (26) and (27) become

(33) $$\begin{array}{cc} P_{\mathrm{tot},N} & =\sum_{j=1}^{N}{\left[\sum_{i=1}^{3n}P\left(w_{i}\right)\right]}_{j}=\sum_{j=1}^{N}{\left[{\left(\sum_{i=1}^{3n}{\hat{w}}_{i}R\left(w_{i}\right)\right)}^{2}\right]}_{j}\; \end{array}$$

(34) $$\begin{array}{cc} J_{\mathrm{tot},N} & =\sum_{j=1}^{N}{\left[\sum_{i=1}^{3n}J\left(w_{i}\right)\right]}_{j}=\sum_{j=1}^{N}{\left[\sum_{i=1}^{3n}w_{i}P\left(w_{i}\right)\right]}_{j}\;. \end{array}$$

Analogously, Equation (28) becomes

(35) $$\begin{array}{cc} v_{\mathrm{tot},N} & =\frac{J_{\mathrm{tot}}}{P_{\mathrm{tot}}}=\frac{\sum_{j=1}^{N}{\left[\sum_{i=1}^{3n}w_{i}P\left(w_{i}\right)\right]}_{j}}{\sum_{j=1}^{N}{\left[\sum_{i=1}^{3n}P\left(w_{i}\right)\right]}_{j}}\;, \end{array}$$

where $w_{i}$ is dependent on the velocities (30) with different $S_{i}$ and $R_{i}$ for every *j*. In quantum mechanical terms the only difference now is that the currents’ nabla operators have to be applied at all of the locations of the respective *N* particles, thus providing

(36) JtotN=∑j=1N1mjReΨ∗t(−iℏ∇j)Ψt

where $\Psi \left(t\right)$ now is the total *N*-particle wave function, whereas the flux lines are given by

(37) vjt=ℏmjIm∇jΨtΨt∀j=1,…,N.

In sum, with our introduction of a relational intensity $P(w_{i})$ for channels $w_{i}$, which include sub-quantum velocity fields, we obtain the guidance formula also for *N*-particle systems in real 3-dimensional space. The central ingredient for this to be possible is to consider the emergence of the velocity field from the interplay of the totality of all of the system’s velocity channels.

**Figure 2:**
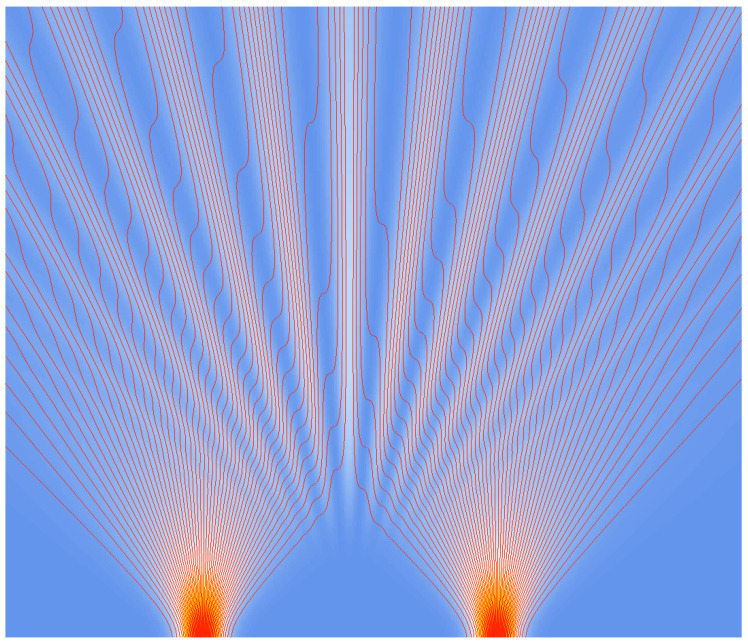
Classical computer simulation of the interference pattern: intensity distribution with increasing intensity from white through yellow and orange, with trajectories (red) for two Gaussian slits, and with *large dispersion* (evolution from bottom to top; $v_{x,1}=v_{x,2}=0$). From [16].

**Figure 3:**
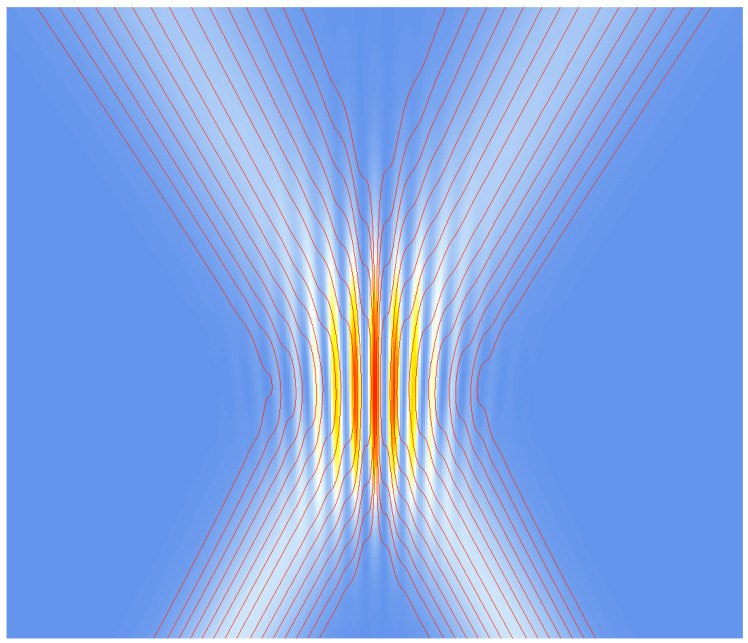
Classical computer simulation of the interference pattern: intensity distribution with increasing intensity from white through yellow and orange, with trajectories (red) for two Gaussian slits, and with *small dispersion* (evolution from bottom to top; $v_{x,1}=-v_{x,2}$). From [16].

In Figure 2 and Figure 3, trajectories (flux lines) for two Gaussian slits are shown (from [16]). These trajectories are in full accordance with those obtained from the Bohmian approach, as can be seen by comparison with [25,26,27], for example.

## 4. Vacuum Landscaping: Cause of Nonlocal Influences without Signaling

In the foregoing sections, we pointed out how nonlocality appears in our model. Particularly in discussing Equations (9)–(11), it was shown that the form of the osmotic momentum

(38) $$mu=-\frac{\hbar }{2}\frac{\nabla P}{P}$$

may be responsible for relevant influences. Moreover, if one assumes a particle at some position x in space, and with a probability distribution *P*, the latter is a distribution around x with long tails across the whole experimental setup, which may be very thin but still non-zero. Then, even at locations y very remote from x, and although the probability distribution *P* pertaining to the far-away particle might be minuscule, it still may become effective immediately through the zero-point field.

The physical reason for bringing in nonlocality is the assumed resonant coupling of the particle(s) with fluctuations of the zero-point vacuum filling the whole experimental setup. Take, for example, a typical “Gaussian slit.” We effectively describe *P* by a Gaussian with long non-zero tails throughout the whole apparatus. As we have seen, in order to calculate on-screen distributions (i.e., total intensities) of particles that went through an *n*-slit device one at a time, one only needs a two-momentum description and a calculation that uses the totality of all relational intensities involving the relative phases determined across the whole apparatus.

In general, we propose a resonant interaction of the bouncing “particle” with a *relevant environment* (In a similar vein, Bohm [28] speaks of a “relatively independent subtotality” of the universe, to account for the possible neglect of the “rest of the universe” in practical calculations). For idealized, non-interacting particles, this relevant environment would be the whole universe and thus the idealized prototype of the “cosmological solution” referred to in the introduction.

For any particle in any experimental setup, however, the relevant environment is defined by the boundary conditions of the apparatus. Whereas the idealized one-particle scenario would constitute an indefinite order of vibrations with respect to the particle oscillations potentially locking in, the very building up of an experiment may represent a dynamical transition from this indefinite order to the establishment of a definite order. The latter is characterized by the emergence of standing waves between the boundaries of the apparatus (e.g., source and detector), to which the particle oscillations lock in. Moreover, if an experimenter decides to change the boundary conditions (e.g., by altering the probability landscape between source and detector), such a “switching” would establish yet another definite order. The introduction or change of boundary conditions, which immediately affects the probability landscape, and the forward and the osmotic fields, we term “vacuum landscaping.”

In other words, the change of boundary conditions of an experimental arrangement constitutes the immediate transition from one cosmological solution in the relevant environment (i.e., within the old boundary conditions) to another (i.e., the new ones). The “surfing” bouncer/particle simply locally jumps from the old to the new standing wave solutions, respectively. This is a process that happens locally for the particle, practically instantaneously (i.e., within a time span ∝1/ω), and nonlocally for the standing waves, due to the very definition of the cosmological solutions. The vacuum landscape is thus nonlocally changed without the propagation of “signals” in a communication theoretical sense (It is *exclusively* the latter that must be prohibited in order to avoid causal loops leading to paradoxes. See Walleczek and Grössing [29,30] for an extensive clarification of this issue).

We have, for example, discussed in some detail what happens in a double-slit experiment if one starts with one slit only, and when the particle might pass it, one opens the second slit [23,31]. In accordance with Tollaksen et al. [32], we found that the opening of the second slit (i.e., a change in boundary conditions) results in an uncontrollable shift in momentum on the particle passing the first slit. Due to its uncontrollability (or, the “complete uncertainty” in [32]), this momentum shift cannot be used for signaling. Still, it is necessary to *a posteriori* understand the final distributions on the screen, which would be incorrect without acknowledging said momentum kick.

Similarly, Aspect-type experiments of two-particle interferometry can be understood as alterations of vacuum landscapes. Consider, for example, the case in two-particle interferometry, where Alice and Bob each are equipped with an interfering device and receive one of the counter-propagating particles from their common source. If Alice during the time-of-flight of the particles changes her device by making with suitable mirrors one of the interferometer arms longer than the other, this constitutes an immediate switching from one vacuum landscape to another, with the standing waves of the zero-point field now reflecting the new experimental arrangement. In other words, the *P*-field has been changed nonlocally throughout the experimental setup and therefore all relational intensities

(39) $$P\left(w_{i}\right)=R\left(w_{i}\right){\hat{w}}_{i}\cdot \sum_{i^{\prime }}{\hat{w}}_{i^{\prime }}R\left(w_{i^{\prime }}\right)$$

involved. The latter represent the relative phase shifts $\delta \varphi_{i,i^{\prime }}=\delta \arccos{\hat{w}}_{i}\cdot {\hat{w}}_{i^{\prime }}$ occurring due to the switching, and this change is becoming manifest also in the total probability density

(40) $$P_{\mathrm{tot}}=\sum_{i}P\left(w_{i}\right)={\left(\sum_{i}{\hat{w}}_{i}R\left(w_{i}\right)\right)}^{2},$$

with *i* running through all channels of both Alice and Bob. The quantum mechanical nonlocal correlations thus appear without any propagation (e.g., from Alice to Bob), superluminal or other. As implied by Gisin’s group [33], this violates a “principle of continuity” of propagating influences from *A* to *B*, but its non-signaling character is still in accordance with relativity and the nonlocal correlations of quantum mechanics. Practically instantaneous vacuum landscaping by Alice and/or Bob thus ensures the full agreement with the quantum mechanical predictions without the need to invoke (superluminal or other) signaling. Our model is, therefore, an example of nonlocal influencing without signaling, which was recently shown to provide a viable option for realistic modeling of nonlocal correlations [29,30].

## 5. Conclusions and Outlook

With our two-momentum approach to an emergent quantum mechanics we have shown that one can in principle base the foundations of quantum mechanics on a deeper level that does not need wavefunctions. Still, one can derive from this new starting point, which is largely rooted in classical nonequilibrium thermodynamics, the usual nonrelativistic quantum mechanical formalism involving wavefunctions, like the Schrödinger equation or the de Broglie–Bohm guiding law. With regard to the latter, the big advantage of our approach is given by the fact that we avoid the troublesome influence from configuration space on particles in real space, which Bohm himself has called “indigestible.” Instead, in our model, the guiding equation is completely understandable in real coordinate space, and actually a rather typical consequence of the fact that the total current is the sum of all particular currents, and the total intensity, or probability density, respectively, is the sum of all relational intensities. As we are working with Schrödinger (i.e., spinless) particles, accounting for differences in particle statistics is still an open problem.

As shown, we can replicate quantum mechanical features exactly by subjecting classical particle trajectories to diffusive processes caused by the presence of the zero point field, with the important property that the probability densities involved extend, however feebly, over the whole setup of an experiment. The model employs a two-momentum approach to the particle propagation, i.e., forward and osmotic momenta. The form of the latter has been derived without any recurrence to other approaches such as Nelson’s.

The one thing that *is* to be digested from our model is the fact that the relational intensities are nonlocally defined, over the whole experimental arrangement (i.e., the “relevant environment”). This lies at the bottom of our deeper-level ansatz, and it is the *only* difference to an otherwise completely classical approach. We believe that this price is not too high, for we obtain a logical, realistic picture of quantum processes which is rather simple to arrive at. Nevertheless, in order to accept it, one needs to radically reconsider what an “object” is. We believe that it is very much in the spirit of David Bohm’s thinking to direct one’s attention away from a particle-centered view and consider an alternative option: that the universe is to be taken as a totality, which, only under very specific and delicate experimental arrangements, can be broken down to a laboratory-sized relevant environment, even if that laboratory might stretch along interplanetary distances. In our approach, the setting up of an experimental arrangement limits and shapes the forward and osmotic contributions and is described as vacuum landscaping. Accordingly, any change of the boundary conditions can be the cause of nonlocal influences throughout the whole setup, thus explaining, e.g., Aspect-type experiments. We argue that these influences can in no way be used for signaling purposes in the communication theoretic sense, and are therefore fully compatible with special relativity.

Accepting that the vacuum fluctuations throughout the universe, or at least within such a laboratory, are a defining part of a quantum, amounts to seeing any object like an “elementary particle” as nonlocally extended and, eventually, as exerting nonlocal influences on other particles. For anyone who can digest this, quantum mechanics is no more mysterious than classical mechanics or any other branch of physics.
